# Mandibular growth in infants with Robin sequence treated with the Tübingen palatal plate

**DOI:** 10.1186/s13005-019-0200-1

**Published:** 2019-06-22

**Authors:** Cornelia Wiechers, Wolfgang Buchenau, Jörg Arand, Anne-Friederike Oertel, Katharina Peters, Silvia Müller-Hagedorn, Bernd Koos, Christian F. Poets

**Affiliations:** 1 Department of Neonatology, Tübingen University Hospital, Calwerstr. 7, D-72076 Tübingen, Germany; 2 Interdisciplinary Center for Cleft Palate and Craniofacial Malformations, Tübingen University Hospital, Tübingen, Germany; 3 Department of Orthodontics, Tübingen University Hospital, Tübingen, Germany; 4 Department of Orthodontics, Rostock University Hospital, Rostock, Germany

## Abstract

**Background:**

Robin sequence (RS) is characterized by mandibular retrognathia, glossoptosis and upper airway obstruction. Whether mandibular catch-up growth may occur in RS is yet controversial. Our functional and less invasive treatment including the Tübingen Palatal Plate (TPP), early oral feeding and orofacial stimulation may promote mandibular catch-up growth. We evaluated the effect of the Tübingen Palatal Plate on mandibular growth, expressed by the Jaw index, sleep study results and weight gain in infants admitted with isolated and syndromic RS, born at or referred to our center between 6/2015 and 5/2018.

**Methods:**

Retrospective analysis of our electronic patient database that included data on jaw index measurements, sleep study results and standard deviation (Z-)scores for weight.

**Results:**

Of 31 patients referred for RS treatment (22 isolated, 9 syndromic), we had data on the above parameters, determined at admission, discharge and 3 months after discharge, in 20. Jaw index at admission and 3-month follow-up was 8.8 (6.3–11.3) and 2.1 (2.0–4.0), respectively (median (IQR); *p* < 0.0001). Mixed-obstructive apnea index (MOAI) decreased from 9.7 (4.8–24.2) to 0.0 (0–1.3; *p* < 0.002). No significant correlation was observed between MOAI and Jaw Index, but MOAI correlated with the Maxillary/Mandibular arch ratio (*r* = 0.58; *p* < 0.001).

Z-scores for weight were similar at both time points at − 1.34 (− 1.76 – − 0.57) and − 1.50 (− 1.89 – − 0.54), while the proportion of infants requiring nasogastric tube feeding decreased from 84 to 8%. No infant had craniofacial surgery; one with syndromic RS required tracheostomy.

**Conclusion:**

These longitudinal cohort data suggest that the Tübingen Palatal Plate as used here may alleviate upper airway obstruction by promoting mandibular growth.

**Trial registration:**

N.A.

## Background

Robin sequence (RS) is characterized by retrognathia, glossoptosis and upper airway obstruction (UAO) with or without cleft palate. Incidence data vary between 1:8500 and 1:14,000 [1, 2]. There are different surgical and conservative treatment approaches to resolve retrognathia and UAO [3, 4], and evidence for UAO to improve with age also using non-surgical treatment [5]. There is controversy, however, whether mandibular growth can be sufficiently promoted to result in disappearance of the maxillomandibular discrepancy seen in RS [6].

Retrognathia can be determined objectively through computed tomography, magnetic resonance tomography, lateral cephalograms, plaster casts, 3-dimensional images, lateral photographs or direct measurements with a measuring tape and caliper [7–9], but there is currently no accepted standard for monitoring retrognathia longitudinally. Most studies assessing mandibular growth used lateral cephalograms at 2 or 3 time points in preschool and school age [9–12], although the largest growth potential of the mandible is probably during infancy. Cephalograms are also not ideal for close monitoring of micrognathia because they involve radiation exposure. Criteria for a simple, non-invasive, widely available, cost-effective and quickly performed method to define and monitor retrognathia in infants are met by the Jaw Index, which can quantify the extent of retrognathia in neonates by means of a measuring tape and a micrometer depth gauge [8]. It is defined as the alveolar overjet X maxillary arch/mandibular arch measured in millimeters. Advantages of the jaw index include its simplicity, applicability as a screening method and suitability for clinical follow-up measurements. However, the index cannot allow conclusions regarding functional or clinical problems and it only identifies retrognathia, which overlaps with, but is not identical to, micrognathia. So far, only few studies have used the Jaw Index to monitor retrognathia longitudinally across several months or years [13]; thus comparing different treatment options is not yet possible.

This may also explain why treatment of UAO in children with Robin sequence varies considerably [14–16]. A recent European survey on current practice patterns showed that two-thirds of clinicians used prone positioning especially in mild RS cases; approaches varied in moderate and severe cases. Non-surgical treatment options included a nasopharyngeal airway (used by 62%) and continuous positive airway pressure (CPAP, used by 45% of respondents). Mandibular distraction and tongue lip adhesion were used by 33 and 18%, respectively, after failure of non-surgical therapy or to avoid tracheostomy. The functional treatment approach developed by our group was used only by some German centers [14].

The latter treatment includes the Tübingen Palatal Plate (TPP), an intra-oral orthodontic appliance with a velar extension, supplemented by early oral feeding and stimulation of the oral musculature based on the Castillo-Morales® approach (for an illustration, see Fig. 1 in ref. [17]); it has been extensively studied in both, mild and severe UAO [18, 19]. In contrast to the results from the above survey [14], we do not consider prone positioning a valid alternative even in mild cases because of its association with an increased risk of Sudden Infant Death Syndrome (SIDS).

Clinically, we observed disappearance of the maxillomandibular discrepancy during the first postnatal months in most infants treated in our center, suggesting mandibular catch-up growth. To assess this more objectively, we introduced a determination of the Jaw Index into our standard clinical protocol upon admission to our center and 3 months after initiation of treatment.

## Patients and methods

### Patients

This was a retrospective audit in all infants with a diagnosis of RS admitted to our center between 6/2015 and 5/2018. This is a national referral center for RS to which most patients are referred by other hospitals after a trial of prone positioning has failed; only a minority is born in our hospital. Clinical data were extracted from the department’s electronic database.

### Treatment protocol

Following hospital admission, infants are monitored in the neonatal intermediate care unit where the severity of UAO is assessed by a multichannel baseline cardiorespiratory sleep study (polygraphy, PG) [16]. Indication for initiating TPP treatment is a mixed-obstructive apnea index (MOAI) > 3 in this initial sleep study.

Next, a maxillary imprint is taken with a custom-made impression tray using alginate (Tetra-Chrom-Super-Alginat, ISO 1563, Klasse B, Typ I, Kanie-Denta, Herford, Germany). This imprint covers the entire hard palate including the cleft, the alveolar ridges and the vestibule. This procedure takes only a few seconds and is carried out in the neonatal intermediate care unit under cardiorespiratory monitoring without sedation, but in the presence of an experienced neonatologist.

The TPP consists of a palatal part that covers the hard palate and the cleft as well as the alveolar ridges and a velar extension of individual length (approximately 3 cm). The shape of this extension is modeled from dental wax and is then attached dorsally to the plaster cast [20]. After an individual prototype of the plate has been produced, infants undergo fiberoptic nasopharyngoscopy without sedation to assess the type and localization of the UAO. During this endoscopy, which usually takes less than a minute, the tip of the extension descending down to the vallecula epiglottica is checked and is ideally located just above the epiglottis. The angle of the velar extension is responsible for the anterior shifting of the base of the tongue and is adjusted so that it pushes the base of the tongue sufficiently forward to erect the epiglottis, thereby widening the pharyngeal space. If the airway appears endoscopically open, the prototype plate is finished and a strengthening wire incorporated into the extension to safeguard the device against mechanical failure. Plates are held in situ with the help of a fixative cream (Corega Super-Haftcreme; Procter & Gamble, Cincinnati, OH) and by extraoral wire bows secured on the infant’s face using adhesive tape (Steri-Strip and Cavilon-No Sting Barrier Film, Steri-Strip Compound Benzoin Tincture, 3 M Health Care, St. Paul, MN, USA). The TPP is worn continuously and its fit regularly controlled by the nursing staff. The plate is briefly removed once daily to clean the alveolar ridge and inspect it for pressure marks or decubitus. After a few days of treatment with a clinically well-fitting TPP its effectiveness in relieving UAO is assessed by a second sleep study, with the aim of achieving a mixed-obstructive apnea index (MOAI) < 3. If this sleep study still shows a MOAI > 3, the plate is modified.

Treatment in infants also comprises appropriate feeding techniques (finger feeding and Playtex Drop-Ins®, Playtex Products, Edgewell, North Bergen, NY, USA) and an orofacial stimulation therapy according to Castillo-Morales®.

The next sleep study is usually performed approximately 3 months following the initial hospital stay. If the palatal part of the plate has become too small, a new TPP is produced and fitted. In general, new plates become necessary because of maxillary growth, i.e. often after approximately 3–4 months or if a notch appears on the alveolar ridges.

### Sleep studies

Cardiorespiratory sleep studies were performed using a computerized polysomnographic system (Embla N 7000, MedCare, Reykjavik, Iceland). The study montage comprised the following channels and sensors: chest and abdominal wall movements (respiratory inductive plethysmography, MedCare), nasal pressure and linearized nasal airflow (nasal prongs and built-in pressure transducer, MedCare), pulse oximeter saturation (SpO_2_) and pulse waveform (Radical, Masimo Inc., Irvine, USA), electrocardiogram (MedCare), and digital video via an infrared camera (Panasonic; Tokyo, Japan). Recordings commenced in the evening and lasted for at least 8 h. All sleep studies were performed in supine position even prior to fitting the TPP, with children being turned to the prone position or recordings terminated if more than 3 desaturations to < 60% SpO_2_ occurred.

Recordings were manually analyzed for the presence of respiratory events as described elsewhere [17, 18, 20]. In brief, total sleep time (TST) was determined from the first to the last 10-min epoch without movement artifact; recordings comprising < 3 h of TST were excluded. Central, mixed and obstructive apneas were identified and a mixed-obstructive apnea index (MOAI) calculated as the sum of mixed and obstructive apneas per hour of TST. Obstructive sleep apnea syndrome (OSAS) was defined as a MOAI > 1.

Desaturation events were defined as a fall in SpO_2_ to < 80% (excluding motion-associated events) and expressed as the desaturation index, defined as events per hour of TST (DI80).

One infant had no sleep study performed because he had arrived at our department already with a tracheostomy in place.

### Determination of the jaw index

**Fig. 1:**
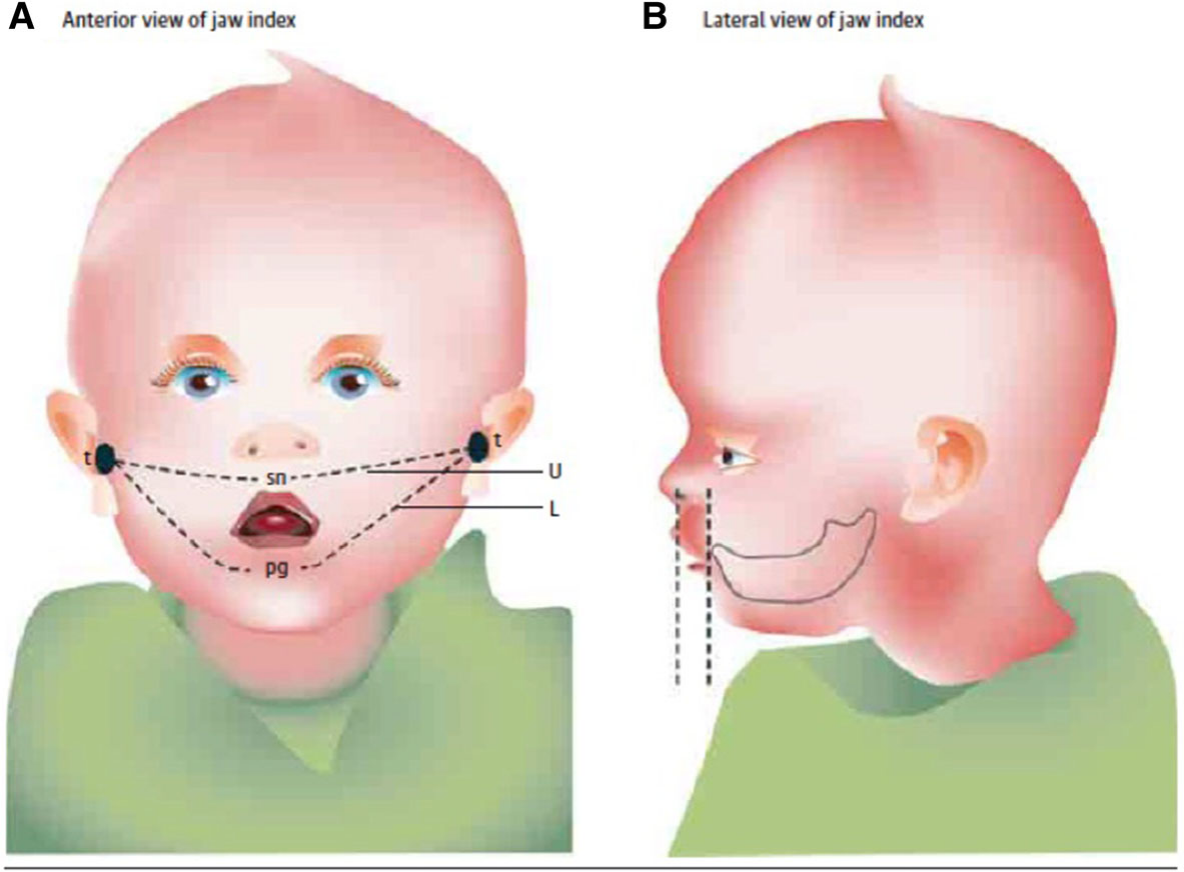
Jaw Index, defined as alveolar overjet (O) x maxillary arch (U)/mandibular arch (L). [Reproduced with permission from JAMA Pediatr. 2016. 170 (9):894-902. Copyright©(2016) American Medical Association. All rights reserved]

The Jaw Index compares the size of upper to that of the lower jaw (Fig. 1). With a small mandible and a clear maxillary overjet, high values are obtained for this index. By using a measuring tape, the maxillary arch was measured from the left to the right tragus via the subnasal point, and the mandibular arch from the left to the right tragus via the pogonion point [8]. The alveolar overjet was determined with a micrometer depth gauge intraorally and defined as the frontodorsal distance between the most anterior points of the upper and lower alveolar arches. The index could usually be measured within < 1 min.

Standard Deviation Scores (SDS) were calulated for weight, length and head circumference

using LMSgrowth (version 2.14; http://www.healthforallchildren.com/?product=lmsgrowth). The reference population was the British 1990 growth reference [21] fitted by maximum penalized likelihood.

### Statistical analysis

Descriptive statistics were used to summarize patient characteristics and sleep study results. Results are reported as median and interquartile range (IQR). Sleep parameters and Jaw Index data obtained upon admission, prior to discharge and 3 months later were compared using the Wilcoxon signed rank test; to assess correlations between MOAI and Jaw Index or Maxillary/Mandibular arch ratio the Pearson correlation coefficient was calculated.

Comparisons of SDS values for weight between various time points were performed using the t-test. To account for the impact of intrauterine growth restriction, SDS differences for weight (SDS_discharge_ − SDS_admission_, SDS_3-month follow-up_ − SDS_admission_) were calculated to illustrate weight gain. Analyses were performed using GraphPad Prism 7 (GraphPad Software, Inc), and the level of significance was *p* < 0.05.

### Ethics

The study protocol was approved by the ethics committee of Tuebingen University Hospital (reference number Pr. Nr. 692/2018BO2).

## Results

### Participants

Thirty-one infants with RS were admitted to our department between 6/2015 and 05/2018; in 20 (65%) complete data including the jaw index were available. Median age at admission was 25 (5.5–48.5) days and at the 3-month follow-up 131.5 (111–177) days. Median duration of the initial hospital stay was 17 (13.5–24) days, ranging from 3 to 61 days. Twenty-two infants (71%) had isolated and 9 (29%) syndromic RS. Demographic and clinical characteristics are summarized in Table 1.

**Table 1:** Patient characteristics

Characteristics	All patients *n* = 31	Patients with Jaw Index data at follow-up *n* = 20^a^
Gender (male/female)	11/20	8/12
Gestational age at birth (weeks)	39.4 (38.4–40.3)	39.0 (38.3–39.7)
5 min APGAR score	9 (7–10)	9 (7–10)
Age at admission (days)	25 (5.5–48.5)	24 (3.8–36.8)
Duration of hospital stay (days)	17 (13.5–24.0)	17 (13.5–24.0)
Weight (g) _admission_	3520 (2988–3993)	3352 (2925–3848)
SDS _admission_	−1.34 (− 1.76 – − 0.57)	−1.29 (− 1.73–0.20)
Weight (g) _discharge_	4130 (3748–4509)	3960 (3658–4361)
SDS _discharge_	−1.14 (− 1.79 – − 0.54)	−1.10 (− 1.79 – − 0.35)
Weight (g) _3 month follow-up_	6093 (5120–6394)	6133 (5350–6391)
SDS _3 month follow-up_	−1.50 (− 1.89 – − 0.54)	−1.61 (− 1.93 – − 0.65)
SDS _admission - birth weight_	−0.90 (− 1.37 – − 0.39)	−0.87 (− 1.32 – − 0.31)
SDS _weight discharge - admission_	− 0.07 (− 0.17–0.30)	− 0.09 (− 0.22–0.39)
SDS _weight 3 month - admission_	− 0.14 (− 0.59–0.59)	− 0.28 (− 0.61–0.38)
Feeding difficulties (NGT) n %		
at admission	26 (84%)	18 (90%)
at discharge	7 (22.6%)	4 (20%)

Values are given as median (IQR), NGT nasogastric tube

^a^All infants with Jaw index data at the 3-month follow-up were treated with a TPP

Upon admission, 10 (32%) infants needed respiratory support: 5 (16%) were treated with continuous positive airway pressure (CPAP) or high flow nasal cannula, 3 with a pharyngeal tube plus CPAP. One infant was intubated and ventilated on admission and one had been tracheotomized by the referring hospital immediately after birth, but had his tracheostomy closed after 2 weeks of TPP treatment. Median MOAI at admission (excluding the infant with a tracheostomy) was 9.7 (4.8–24.2) and median DI80 was 0.14 (0–1.7; Table 2). Three infants (9.7%) had no OSAS and were only treated with a conventional palatal plate to cover their cleft, all other infants were treated by TPP. No infant needed mechanical ventilation at discharge, but one with syndromic RS did not benefit from TPP treatment and required a tracheostomy. The infant admitted with a tracheostomy had no initial sleep study; four infants did not have a sleep study at discharge (one required a tracheostomy, and three had no OSAS upon admission and were only treated with a conventional palatal plate). All remaining infants had at least two interpretable sleep studies recorded, one prior to and one following treatment. All patients reported in the 3-month follow-up group were treated with a TPP.

**Table 2:** Longitudinal changes in MOAI and DI80 during treatment

Variables	Admission *n* = 30	Prior to discharge *n* = 27	3-month follow-up *n* = 26
Sleep study			
MOAI (events/h)	9.7 (4.8–24.2)	0.4 (0.1–1.2)	0.0 (0–1.3)
DI80 (events/h)	0.14 (0–1.7)	0 (0–0)	0 (0–0)

Values are given as median (IQR); *MOAI* mixed-obstructive apnea index, *DI80* desaturation index

There were no serious adverse events like systemic infections or pulmonary aspiration. Predominant side effects were temporary pressure marks/decubitus on the hard or soft palate, which all healed within a few days after manually reshaping the plate.

SDS-scores for weight were similar at admission (− 1.34 (− 1.76 – − 0.57)), discharge (− 1.14 (− 1.79 – − 0.54; *p* = 0.97)) and at the 3-month follow-up (− 1.50 (− 1.89 – − 0.54; *p* = 0.68)).

At discharge, 7 infants (23% of *n* = 31) still received nasogastric tube feedings; this proportion fell to 2 infants with syndromic RS (8% of *n* = 26) at the 3-month follow-up.

Average value of the Jaw Index at admission was 8.8 (6.3–11.3), compared to 2.1 (2.0–4.0; *p* < 0.0001) at the 3-month follow up, the latter data in line with reference data on healthy infants obtained previously (Table 3).

**Table 3:** Jaw Index measurements at birth compared to published data in controls

Measure	Tübingen RS		Vegter et al. [13] RS		Vegter et al. [13] Controls	
	*n* = 31 at admission	*n* = 20^a^ at 3 months follow	*n* = 7 at birth	*n* = 7 at 0.5 yr	*n* = 100 at birth	*n* = 42 at 0.5 yr
Maxillary arch (mm)	168.0 (160–179)	185.6 (181–199)	162.4 (11.6)	193.0 (3.3)	167.9 (12.3)	206.0 (8.7)
Mandibular arch (mm)	152.0 (142.4–161.3)	181.1 (177–189)	146.0 (11.4)	176.3 (7.6)	159.4 (12.0)	207.5 (8.3)
Maxillary/ Mandibular arch	1.10 (1.06–1.13)	1.04 (1.00–1.06)	1.11	1.09	1.05	0.99
Overjet, (mm)	8.8 (6–10)	2.3 (2.0–4.0)	10.8 (2.7)	6.5 (2.6)	4.0 (1.7)	2.0 (0.8)
Jaw Index, (mm)	8.8 (6.3–11.3)	2.1 (2.0–4.0)	12.1 (3.2)	7.2 (2.9)	4.2 (1.8)	2.0 (0.8)

Values are given as median IQR and mean and SD (Vegter et al. [13])

^a^All infants with Jaw index data at the 3-month follow-up were treated with a TPP

**Fig. 2:**
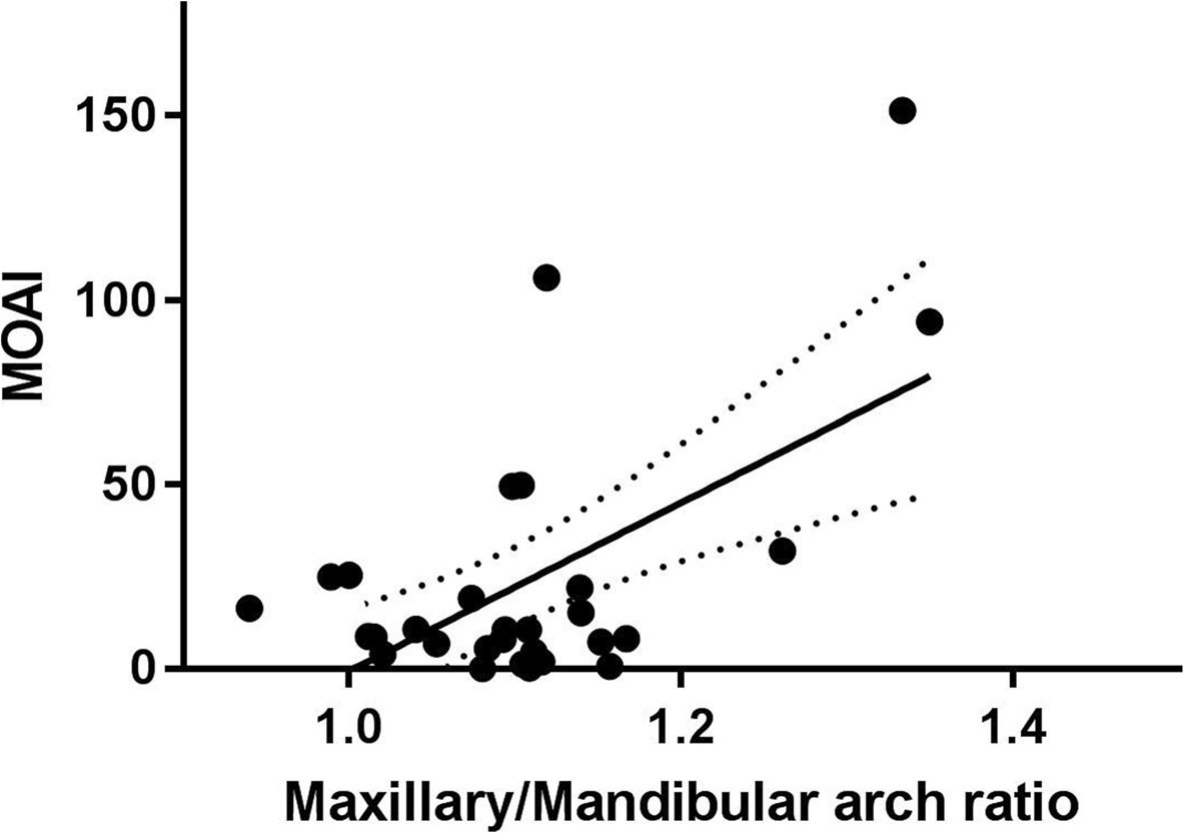
Correlation between the Maxillary/Mandibular arch ratio and the mixed-obstructive apnea index in study infants

Correlation analysis according to Pearson showed no significant correlation between MOAI and Jaw Index, but between the former and the maxillary/mandibular arch ratio (*r* = 0.58; *p* = 0.006; Fig. 2). Linear regression showed that an increase in the Maxillary/Mandibulary ratio of 0.1 led to an increase in the MOAI of approximately 20.

There were no differences in sleep study or jaw index results between infants with isolated vs. syndromic RS, but feeding difficulties were more common in the latter (Table 4).

**Table 4:** Patient characteristics, Jaw Index and sleep study results in isolated and syndromic RS

	Isolated RS *n* = 22	Syndromic RS *n* = 9
SDS for weight		
Admission- birth weight	−1.21 (− 1.42 – − 0.24)	0.68 (0.90 – − 0.43)
Discharge-admission	−0.1 (− 0.21–0.38)	−0.06 (− 0.09–0.11)
Weight 3 month-admission	−0.12 (− 0.4–0.76)	−0.58 (− 1.43–0.33)
Feeding difficulties (NGT) n (%)		
at admission	16/22 (73%)	9/9 (100%)
at discharge	3/22 (14%)	4/9 (44%)
at 3 month follow-up	0/19 (0%)	2/7 (29%)
Jaw Index (mm)		
at admission	9.0 (7.0–11.3)	6.8 (4.2–9.1)
at 3 month follow-up	2.6 (2.1–4.1), *n* = 15^a^	2 (1.3–2.1), *n* = 5^a^
MOAI (events/h)		
at admission	10.6 (5.1–31.5)	8.8 (5.5–15.2)
at discharge	0.3 (0.1–1.0)	0.9 (0.1–2.1)
at 3 month follow-up	0 (0–0.9), *n* = 19	0.2 (0–1.6), *n* = 7

^a^All infants with Jaw index data at the 3-month follow-up were treated with a TPP

## Discussion

In this single-center cohort of infants referred with RS, we confirmed that a combination of TPP, orofacial stimulation and feeding training reduced sleep-related UAO and improved feeding problems [17, 22], and that this was also associated with less micrognathia, as expressed by the jaw index, within 3 months of treatment onset. These findings suggest that mandibular catch-up growth may indeed be taking place during TPP treatment in RS infants.

Studies on mandibular growth in RS patients with long-term follow-up after conservative management are rare, and most involved only small patient numbers [9–12, 23–26]. Two of eight longitudinal studies supported the concept of mandibular catch-up growth [12, 25], while the remaining reported rates of sagittal mandibular growth similar to controls. Controls were either healthy children or those with cleft palate, thus there were likely also growth differences between the various control groups in these studies, and most used lateral cephalograms [27] to assess the facial profile at 2 or 3 time points at preschool or school age [9–12], i.e. when the growth potential of the mandible is lower.

In contrast, the jaw index affords a simple, cost-effective method to assess retrognathia during childhood without any radiation exposure [8, 13] and can thus be used as a screening tool that is also suitable for clinical follow up measurements. Vegter et al. used the Jaw Index for longitudinal measurements in seven infants with RS that were compared to healthy controls studied at birth and at 6 and 12 months [13]. Five of these RS patients were treated with tongue-lip adhesion. During the 1-year study period, the jaw index decreased from 4.2 (0.0–9.5) to 1.5 (0.94–3.1) in controls, and from 12.1 (6.5–13.8) to 4.3 (2.2–7.7) in RS patients [13]. Even so, it remained higher than in controls, and proportional mandibular growth was similar to controls, so that catch-up growth could not be confirmed to occur in their study.

The Jaw Index, however, does not allow any conclusions regarding functional or clinical aspects. In our patient group, we were unable to establish a correlation between Jaw Index and sleep study results like the MOAI. Interestingly, however, we found a significant correlation between the MOAI and the maxillary-to-mandibular ratio. Provided this parameter has sufficient reproducibility and interobserver variability, it may be more suitable to assess treatment success in infants with RS. Furthermore, depending on the activity of the infant, it is our experience that it can at times be difficult to determine the alveolar overjet using a micrometer depth gauge.

Only few studies investigated longitudinal changes in the Jaw Index in retrognathic patients [13], thus comparing different therapeutic options is currently not possible. Another difficulty is that there are yet no reliability studies to assess intra- and interobserver variability for this parameter.

An explanation for the mandibular catch-up growth seen here after TPP treatment may be provided by the “form follows function” paradigm. The TPP aims to push the base of the tongue forward to widen the hypopharynx. In doing so, it shifts the mandible into a more anterior position, thereby stimulating condylar growth, which enables a skeletal adaptation to this new mandibular position. Such histological changes in the condylar cartilage of the temporomandibular joint have already been described in detail for functional orthodontic appliances in a rhesus monkey model [28], but still require confirmation by further studies.

We have documented the persistence of normal sleep study results up to 3 months after hospital discharge.

A large proportion of our patients (29%) had evidence of an associated syndrome, as also reported by others [29, 30]. The potential of mandibular growth in these patients may not be the same as in those with isolated RS [31]. Thus, we considered it encouraging that there were no apparent differences in mandibular growth between infants with isolated RS vs. syndromic patients, but again, numbers were small.

Additional limitations include the retrospective nature of our study and missing values for the jaw index in a significant proportion of children at the 3-month follow-up. The latter was due to difficulties in scheduling these measurements for infants living far away. We do not know whether the catch-up growth seen was a treatment effect or occurred spontaneously, as we did not include a control group of untreated RS infants. All infants with Jaw Index data at the 3-month follow-up were treated with the TPP.

Only 7% of respondents to a recent European survey on current practice patterns used the Jaw Index as a diagnostic criterion [14], and to our knowledge there is only one study that used the Jaw Index to monitor retrognathia longitudinally over the first months of life [13]. Therefore, a comparison of Jaw Index measurements over time between non-surgical or surgical treatment options is not available.

## Conclusion

Our results suggest that mandibular catch-up growth may occur in RS infants treated with the TTP, which should now be confirmed prospectively. A comparison of Jaw Index data with other treatment strategies, e.g. in the context of a cross-national clinical database for Robin infants, would be desirable. Whether another non-invasive method for determining retrognathia and assessing mandibular growth longitudinally, for example standardized 2D or 3D photos, is helpful in this regard, should also be investigated in further studies.

## Data Availability

Data sharing is not applicable to this article as no datasets were generated or analysed during the current study.

## Abbreviations

CPAP: Continuous positive airway pressure
DI80: Desaturation index
MOAI: Mixed obstructive apnea index
N/A: Not available
OSAS: Obstructive sleep apnea syndrome
RS: Robin Sequence
SD: Standard deviation
SDS: Standard deviation score
SIDS: Sudden Infant Death Syndrome
TST: Total sleep time
TPP: Tuebingen Palatal Plate
UAO: Upper airway obstruction
